# Socioeconomic status and metabolic syndrome in Southwest Iran: results from Hoveyzeh Cohort Study (HCS)

**DOI:** 10.1186/s12902-022-01255-5

**Published:** 2022-12-28

**Authors:** Nader Saki, Seyed Jalal Hashemi, Seyed Ahmad Hosseini, Zahra Rahimi, Fakher Rahim, Bahman Cheraghian

**Affiliations:** 1 Hearing Research Center, Clinical Sciences Research Institute, Department of Otolaryngology, Head and Neck Surgery, Ahvaz Jundishapur University of Medical Sciences, Ahvaz, Iran; 2 Department of Internal Medicine, School of Medicine, Alimentary Tract Research Center, Clinical Sciences Research Institute, Ahvaz Jundishapur University of Medical Sciences, Ahvaz, Iran; 3 Nutrition and Metabolic Diseases Research Center, Clinical Sciences Research Institute, Ahvaz Jundishapur University of Medical Sciences, Ahvaz, Iran; 4 Department of Biostatistics and Epidemiology, School of Public Health, Ahvaz Jundishapur University of Medical Sciences, Ahvaz, Iran; 5 Thalassemia and Hemoglobinopathy Research Center, Research Institute of Health, Ahvaz Jundishapur University of Medical Sciences, Ahvaz, Iran; 6 Alimentary Tract Research Center, Clinical Sciences Research Institute, Ahvaz Jundishapur University of Medical Sciences, Ahvaz, Iran

**Keywords:** Socioeconomic status, Metabolic Syndrome, PERSIAN Cohort, Iran, Hoveyzeh

## Abstract

**Background:**

Socioeconomic status (SES) strongly predicts morbidity and premature mortality, especially for non-communicable diseases (NCD**s**). However, the effect of these factors on Metabolic Syndrome (MetS) is not clear yet. This study was conducted to assess the relationship between socioeconomic indicators and MetS.

**Methods:**

In this prospective cohort study, 10,009 people aged 35–70 enrolled from May 2016 to August 2018. The MetS was defined according to The Standard National Cholesterol Education Program (NCEP)—adult treatment panel III (ATP III) or NCEP-ATP III criteria. Demographics and socioeconomic data were gathered face-to-face through trained interviews. Also, lab, anthropometrics, and blood pressure measurements were assayed for participants. Logistic regression was used to estimate the association between SES and MetS, adjusted for the potential confounding factors.

**Results:**

The overall prevalence of MetS in the participants was 39.1%. The crude odds ratios were statistically significant for all the assessed variables (*p* < 0.05). After adjustment for age, sex, physical activity, smoking, and alcohol use as potential confounders, the results indicated significant direct independent associations between skill level (*p* = 0.006) and Townsend index (*p* = 0.002) with MetS. In contrast, no significant associations between educational level and wealth status with MetS.

**Conclusion:**

The results of our study showed that SES is related to MetS. Among the four assessed SES indicators, skilled levels and Townsend score are strongly associated with MetS. We recommend considering people's SES when interventional programs are planned and conducted on MetS in similar communities.

**Supplementary Information:**

The online version contains supplementary material available at 10.1186/s12902-022-01255-5.

## Background

Non-communicable diseases are the leading global reason for death and excessively afflict those living in low-income and lower-middle-income countries (LLMICs) [1]. Almost three-quarters of deaths and 82% of premature deaths occur within LLMICs due to non-communicable diseases (NCD**s**) [2]. Generally, morbidity and mortality rates of diseases are higher among people with lower socioeconomic status (SES) [3]. The primary aim of social epidemiology is to study the relationship between SES and population health outcomes. Today, it is well known that in almost all countries, more people of lower SES have experienced poorer health than those of higher SES [4].

Metabolic syndrome (MetS) combines abdominal obesity, glucose intolerance, dyslipidemia, and hypertension. These disorders are well-known risk factors for cardiovascular disease and type 2 diabetes [5]. The prevalence of MetS has been estimated at around 20–25% of the world's adult population [6]. It has rapidly increased in developed and developing countries [7–9]. Socioeconomic factors are actively involved in the development of MetS. Some studies conducted in the United States [10], Netherlands, Poland [11], Tunis [12], Suriname [13], Iran [14, 15], Brazil [16–18], and Mesoamerican countries [19] showed that MetS prevalence decreases with better education [20–22], occupational class [23], income [24, 25], and wealth status [26]. On the other hand, reverse patterns were observed in Nigeria, Saudi Arabia, and India, i.e., there was more likelihood for individuals with high socioeconomic status for having MetS [27–29].

Due to the higher prevalence rate of MetS in Hoveyzeh [30], compared to the rates of other regions of Khuzestan province rates [31] and Iran [32], and also the important role of SES in NCDs, we designed a study to investigate the relationship between metabolic syndrome and socioeconomic factors, including education, wealth index, skill level, and Townsend deprivation index in the context of a well-designed population-based cohort study.

## Methods

### Study design and sampling method

The Hoveyzeh Cohort Study (HCS) is a population-based cohort study designed to assess NCDs in southwest Iran [33]. HCS is one of the sites of the Prospective Epidemiological Research Studies in IrAN (the PERSIAN Cohort Study) [34] and recruited 10,009 adults (age 35–70 years) from May 2016 to August 2018. All data and measurements in the PERSIAN cohort sites (18 regions with different ethnicities) were collected according to the same protocol, the Standard instrument. Based on the 2016 door-to-door census, 12,103 eligible individuals lived in the Hoveyzeh district. Invitations to the cohort site were given by trained inviters one week before the referral day. A phone call was made to remind the invitees the day before the visit. Out of 12,103 eligible individuals invited, 8792 were enrolled in the study for the first stage, 982 for the second stage, and 235 for the third invitation stage. Finally, 10,009 individuals entered the study. The overall response proportion was 85.16%. Inclusion criteria consisted of: (1) age of 35–70 years old, (2) residence in Hoveyzeh, (3) lack of severe mental disorder and ability to answer the questionnaires without help, (4) Not to be deaf and dumb. We excluded participants without data on SES or missing data for MetS.

### Definition of the MetS

The criteria for MetS diagnosis were: 1) abdominal obesity (waist circumstance ≥ 102 in men and ≥ 88 in women), 2) high serum triglycerides (≥ 150 mg/dL) or taking hypertriglyceridemia medications, 3) abnormal serum high-density lipoprotein (HDL) cholesterol (≥ 40 mg/dL in men and NCDs 50 in women), or take drug treatment for low HDL cholesterol,4) high blood pressure ≥ 130/85 mmHg, or take hypertension drugs, 5) high fasting plasma glucose (FPG) ≥ 100 mg/dL, or take hyperglycemia drugs. The presence of at least 3 out of the 5 mentioned criteria used in the case definition constituted a diagnosis of MetS [30].

### Components of MetS measurements and quality control of laboratory

Individuals attending the study had been fasting for about 10 to 12 h on the day of enrollment. 27 cc of blood was taken from each participant. Tubes clot were placed at room temperature for 30 to 40 min before centrifugation under a Class II laminate laboratory hood. The serum was separated from the rest of the blood during this time. Then, the clot tubes were located in the centrifuge(Sigma, Germany) at 3000 rpm for 10 to 15 min. The required serum levels were measured by BT 1500 autoanalyzer (Biotecnica Instruments, Italy). Normal and pathogen control serum samples were defined and RUN for BT 1500 device. Finally, control serum results were evaluated in Westgard and Levy Jennings quality control chart. From these data, the mean and SD were calculated. Levy Jennings chart was constructed with x + 2 SD as warning limits and x + 3 SD as control limits. The percent coefficient of variation (CV) was defined as SD times 100 divided by the mean value of the results in a set of replicate measurements. Therefore, a smaller CV indicates higher precision. The precision data obtained for routine biochemical analytes by BT 1500 autoanalyzer consist of HDL (Mean 30.8, CV% 2.75), Cholesterol (Mean 152, CV% 2.70), TG (Mean 110, CV% 2.28), FBS (Mean 94.4, CV% 2.71). Quantitative diagnostic kits for biochemical tests of Cholesterol, TG(GPO-PAP), HDL(IMMUNO), and FBS were from Pars Azmoun company in Iran and used the BT 1500 autoanalyzer. (Biotecnica Instruments, Italy).

### Anthropometric measurements

Anthropometric measurements were taken by trained staff. Height(cm) was measured by a Stadiometer (Seca 206) in a standing position without shoes, shoulders relaxed, facing forward with head and back facing the wall. Weight (kg) was measured with minimal clothing on a standing scale (Seca 755). Also, a locked tape meter (Seca) was used for measuring the waist, wrist, and hip circumference (cm).

### Blood pressure measurements

At least half an hour before blood pressure measurement, participants should not exercise, have heavy physical activity, have not consumed heavy food, coffee, alcohol, drugs, and stimulant drinks, and have not smoked. Before measuring the first blood pressure, the participant must rest for 1–2 min. A Richter sphygmomanometer with a suitable cuff size was used. The blood pressure cuff was neither tight nor too tight on the bare arm. Blood pressure was measured from the right and left arms of the person twice at a ten minutes interval, and when measuring blood pressure, the person's hand was placed on a flat surface such as a table.

### SES indicators

We used four indicators to assess SES in this analysis: The Townsend deprivation index, as an area-level indicator of SES, the wealth index, as a household-level indicator of SES, and educational level and skill level as individual-level socioeconomic indicators.

The wealth index was calculated according to the information on households’ assets, including freezer, TV, motorbike, cell phone, car, vacuum cleaner, internet access, washing machine, computer, and household utilities consisting of house ownership, and the number of rooms per capita. A principal component analysis (PCA) was conducted to assign a coefficient to each asset. The sum of the first component scores constructed the wealth scores. Eventually, the scores were converted into 5-ordered categories, including poorest, poor, moderate, rich, and richest, based on the quintiles [35].

Townsend deprivation index was calculated in four steps: 1) To calculate the percentage of households with non-car ownership, non-house ownership, having unemployed adults, and overcrowding; 2) To calculate logged unemployed and logged overcrowds; 3) To calculate the Z score of no car, non-homeowners, unemployed, and overcrowd; 4) To calculate Z score of no car + Z score of the non-homeowner + Z score of unemployed + Z score of overcrowding = TDS. Finally, the calculated scores were categorized into five ordinal categories Based on the quintiles, including most affluent, affluent, moderate, deprived, and most deprived [36].

In our study, occupational classification was done according to the International Standard Classification of Occupations (ISCO-8). Skill level is a function of the complexity and range of tasks and duties related to an occupation. Four broad and ordered skill levels are used in ISCO-08. Skill Level 1 typically involves performing simple and routine physical or manual tasks. The people categorized in Skill Level 1 may require physical strength and/or endurance. In skill level 2, reading information and performing simple arithmetical calculations are usually essential. Skill level 3 generally requires a high level of literacy, numeracy, and well-developed interpersonal communication skills. Finally, Skill level 4 requires extended levels of literacy and numeracy, sometimes at a very high level, and excellent interpersonal communication skills [37].

### Lifestyle measurements

We used the International Physical Activity Questionnaire (IPAQ) [38] to measure the participants' physical activity levels by the metabolic equivalent of the task (MET Index). That is the ratio of a person's working metabolic rate relative to his/her resting metabolic rate. One MET is the energy cost of sitting quietly and is equivalent to a caloric consumption of one kcal/kg/hour. A person who has smoked no less than 100 cigarettes during his or her lifetime is defined as a smoker. To determine alcohol abuse among participants, we asked whether they have continually used them in their lifetime. The consumption amount and its type have also been asked [1].

### Statistical analysis

Descriptive statistics measures were performed using mean and standard deviation for quantitative variables, while frequency and percentage were used for categorical variables. The Chi-square test, chi-square test for trend, and crude odds ratio were used to assess univariate analysis associations. The effect of covariates (Age, Sex, Educational level, Current Smoking, Alcohol use, and MET) on the assessed relationships were controlled using a multiple logistic regression model. Adjusted odds ratios were obtained from this model. All reported *p*-values were based on two-tailed tests and compared to a significance level of 0.05. IBM® SPSS® Statistics 26.0 was used for the statistical analysis.

## Results

Among 10,009 participants, 59.8% (*n* = 5983) were female. The mean ± SD of the participants’ age was 48.8 ± 9.2 years. The overall prevalence of MetS in participants was 39.1% (95% CI 38.1 – 40.1). In general, the prevalence of MetS was higher in women (45.7%) compared to men (29.3%) (*p* < 0.001). The prevalence rates, in total, were raised by increasing the age of the participants, although, in males, the rates slightly decreased over the age of 60 years (Table S1).

Bivariate analysis in Table 1 showed that the prevalence of MetS was associated with age, sex, educational level, MET, wealth status, job, Townsend index, smoking, and alcohol use (*P* < 0.05). The prevalence of MetS was higher among females, smokers, and non-alcoholic people than the others (*P* < 0.05). Also, the prevalence of MetS was significantly higher among older than younger people. The result of trend analyses indicates an upward pattern significantly for the age group (*P* < 0.001) and wealth status (*P* = 0.041), while this pattern was downward for educational level, area deprivation status (Townsend Index), and physical activity (*P* < 0.001). Besides, the lowest prevalence of MetS was seen among participants in Skill Level IV (mainly manual workers), although the trend was not significant (*P* for trend = 0.48).

**Table 1:** Prevalence of MetS by demographic, socioeconomic, and lifestyle characteristics of study participants

Variable		Total	Cases	Prevalence % (CI 95%)	*P*-value *	*P*-value for trend**
Overall prevalence		10,009	3913	39.1 (38.1 – 40.1)		
Age Group	35–39	1912	475	24.8 (22.9 – 26.8)	< 0.001	< 0.001
	40–44	2025	646	31.9 (29.9 – 34.0)		
	45–49	1797	716	39.8 (37.6 – 42.2)		
	50–54	1482	672	45.3 (42.8 – 47.9)		
	55–59	1281	641	50.0 (47.3 – 52.8)		
	60–64	798	391	49.0 (45.5 – 52.5)		
	≥ 65	714	372	52.1 (48.4 – 55.8)		
Sex	Male	4026	1180	29.3 (27.9 – 30.7)	< 0.001	
	Female	5983	2733	45.7 (44.4 – 47.0)		
Educational level	Illiterate	6209	2654	42.7 (41.5 – 44.0)	< 0.001	< 0.001
	Primary school	1665	599	36.0 (33.7 – 38.3)		
	Secondary school	673	225	33.4 (29.9 – 37.1)		
	High school	741	232	31.3 (28.0 – 34.8)		
	University	721	203	28.2 (24.9 – 31.6)		
Physical activity (MET Score)	Q1	2503	1201	48.0 (46.0 – 50.0)	< 0.001	< 0.001
	Q2	2505	1053	42.0 (40.1 – 44.0)		
	Q3	2508	913	36.4(34.5 – 38.3)		
	Q4	2493	746	29.9(28.1 – 31.8)		
Wealth Status	Poorest	2012	748	37.2 (35.1 – 39.3)	0.003	0.041
	Poor	2057	750	36.5 (34.4 – 38.6)		
	Moderate	2087	846	40.5 (39.7 – 44.0)		
	Rich	2023	838	41.4 (39.3– 43.6)		
	Richest	1830	731	39.9 (37.7 – 42.2)		
Job	Skill Level I	290	50	17.2 (13.1 – 22.1)	< 0.001	0.48
	Skill Level II	2561	761	29.7 (27.9 – 31.5)		
	Skill Level III	84	28	33.3 (23.4 – 44.5)		
	Skill Level IV	345	88	25.5 (21.0 – 30.5)		
Area deprivation status (Townsend Index)	Most Affluent	2390	1190	49.8 (47.8 – 51.8)	< 0.001	< 0.001
	Affluent	1856	728	39.2 (37.0 – 41.5)		
	moderate	1890	781	41.3 (39. 1 – 41.6)		
	Deprived	1293	425	32.9 (30.3 – 35.5)		
	Most Deprived	2580	789	30.6 (28. 8 – 32.4)		
Smoking	Yes	7920	3191	40.3(39. 2 – 41.4)	< 0.001	
	No	2089	722	34.7(32. 5 – 36.6)		
Use Alcohol	Yes	197	61	31.0(24. 6 – 37.9)	0.019	
	No	9812	3852	39.3 (38. 3 – 40.2)		

^*^Chi-square test

^**^Chi-square for trend test

### Multivariable analysis

The crude and adjusted odds ratios using the logistic regression model are presented in Table 2. For all the assessed variables, the crude odds ratios were statistically significant (*p* < 0.05); therefore, we included them in the multiple logistic regression model. According to the Hosmer–Lemeshow test results, the model's goodness of fit was acceptable (Chi-square = 9.67, df = 8, *P* = 0.29). After adjusting for age, sex, physical activity, smoking, and alcohol use as potential confounders, the results indicated independent, direct significant associations between skill level and Townsend index with MetS. In contrast, no significant associations were seen between educational level and wealth status with the condition.

**Table 2:** The crude and adjusted odds ratios of the assessed factors for MetS and their 95% confidence interval using the logistic regression model

Variable		Crude Odds Ratio (95% CI)	*Adjusted Odds Ratio (95% CI)	*P*-value
Age Group	35–39	1	1	< 0.001
	40–44	1.42 (1.23–1.63)	1.33 (1.04. – 1.68)	
	45–49	2.00 (1.74 – 2.31)	1.79 (1.40 – 2.30)	
	50–54	2.51 (2.17 – 2.90)	1.99 (1.51 – 2.61)	
	55–59	3.03 (2.61 – 3.52)	1.78 (1.31 – 2.43)	
	60–64	2.91 (2.44 – 3.46)	1.74 (1.16 – 2.60)	
	≥ 65	3.29 (2.75 – 3.94)	2.02 (1.24 – 3.27)	
Sex	Male	1	1	< 0.001
	Female	2.028 (1.86– 2.21)	1.87 (1.43 – 2.44)	
Educational level	Illiterate	1	1	0.065
	Primary school	0.75 (0.67 – 0.84)	1.24 (0.99 – 1.54)	
	Middle school	0.67 (0.57 – 0.80)	1.36 (1.04 – 1.78)	
	High school	0.61 (0.52– 0.72)	0.96 (0.73 – 1.27)	
	University	0.53 (0.44 – 0.62)	1.04 (0.75 – 1.44)	
Current Smoking	No Smoker	1	1	0.81
	Smoker	1.28 (1.15 – 1.41)	0.98 (0.82 – 1.017)	
Alcohol use	No	1		0.42
	Yes	.69 (.51-.94)	1.17 (0.81 – 1.69)	
MET	Q1	2.16 (1.92–2.43)	1.57 (1.26–1.96)	< 0.001
	Q2	1.70 (1.51–1.91)	1.31 (1.05–1.64)	
	Q3	1.34 (1.19–1.51)	1.08 (0.87–1.35)	
	Q4	1	1	
Wealth Status	Poorest	1	1	0.30
	Poor	0.97 (0.85 – 1.10)	1.13 (0.83 – 1.54)	
	Moderate	1.15 (1.02 – 1.31)	1.25 (0.92 – 1.69)	
	Rich	1.19 (1.05 – 1.36)	1.21 (0.89 – 1.65)	
	Richest	1.12 (0.99–1.28)	1.40 (1.01–1.93)	
Job	Skill Level I	1	1	0.006
	Skill Level II	2.03 (1.48 – 2.78)	1.57 (1.13 – 2.18)	
	Skill Level III	2.40 (1.39 – 4.14)	1.89 (1.07 – 3.35)	
	Skill Level IV	1.64 (1.11–2.43)	1.09 (0.70 – 1.76)	
Townsend Index	Most Affluent	2.25 (2.01–2.53)	1.71 (1.26 – 2.31)	0.002
	Affluent	1.46 (1.29 – 1.66)	1.23 (1.01 – 1.51)	
	Moderate	1.60 (1.41 – 1.81)	1.49 (0.72 – 2.47)	
	Deprived	1.11 (0.96 – 1.28)	1.29 (1.02 – 1.63)	
	Most Deprived	1		

^*^The adjusted odds ratios are controlled for age, sex, physical activity, smoking, and alcohol use

**Fig. 1:**
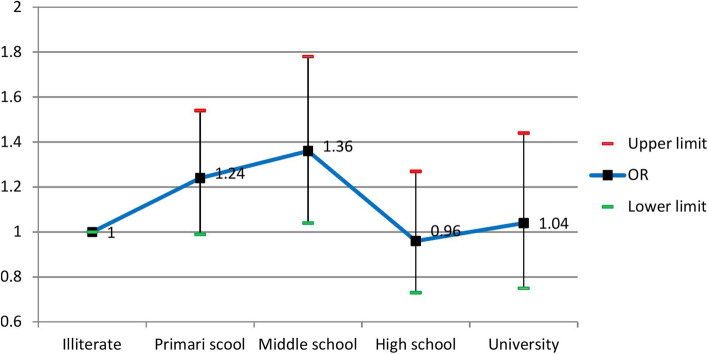
Adjusted ORs (95% CI) of MetS related to levels of education

**Fig. 2:**
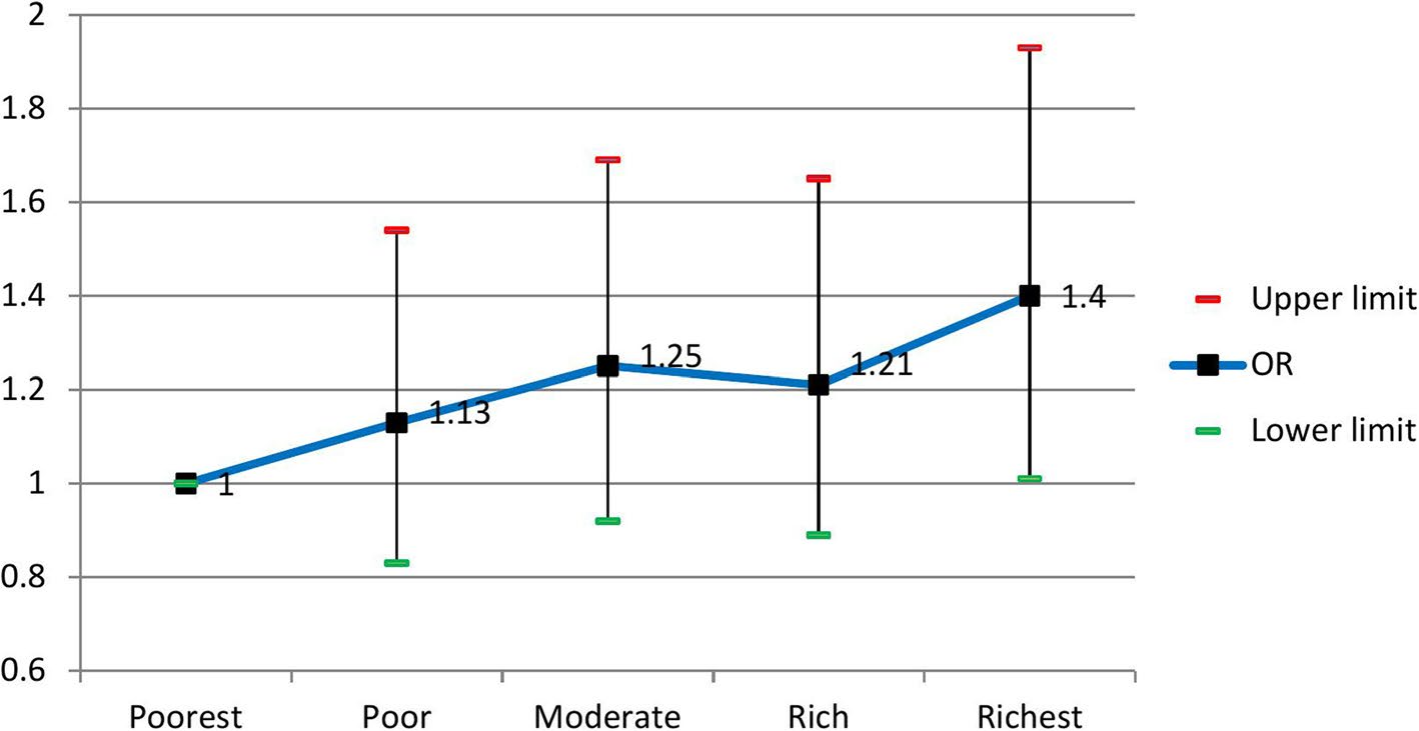
Adjusted ORs (95% CI) of MetS related to wealth status

**Fig. 3:**
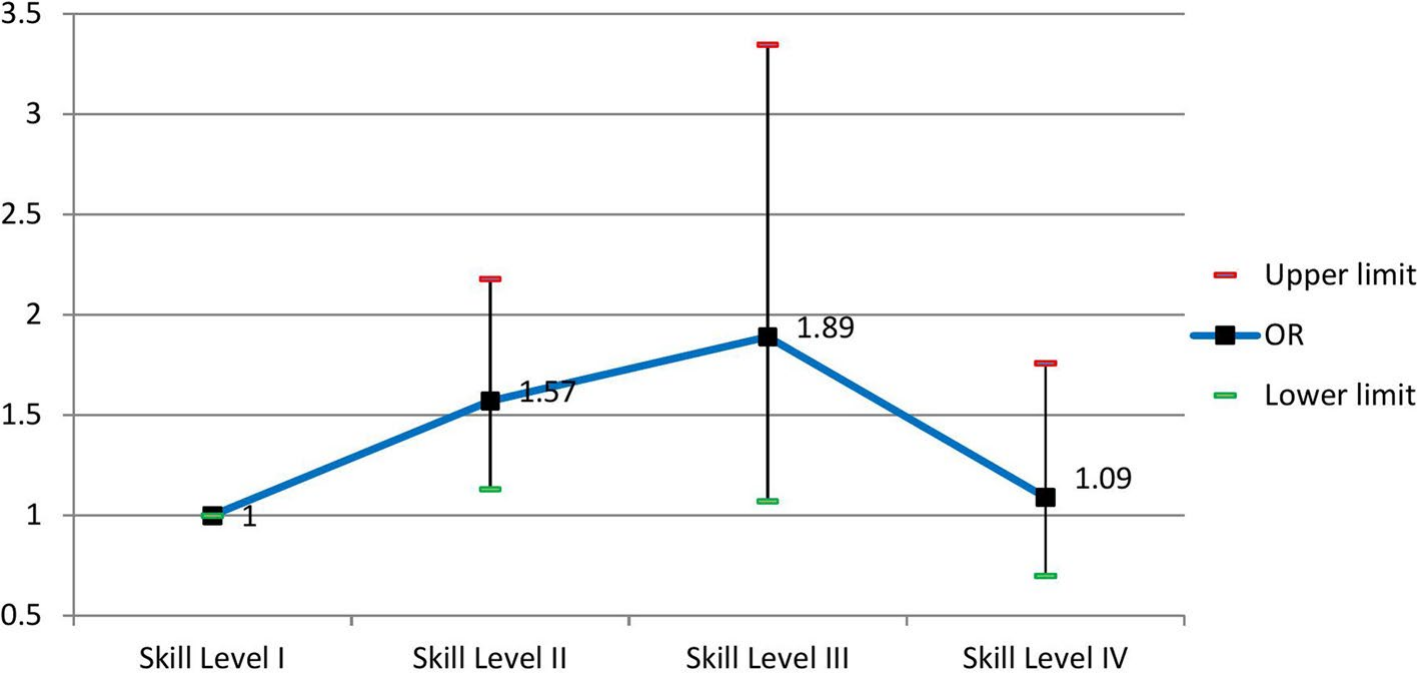
Adjusted ORs (95% CI) of MetS related to skill levels

**Fig. 4:**
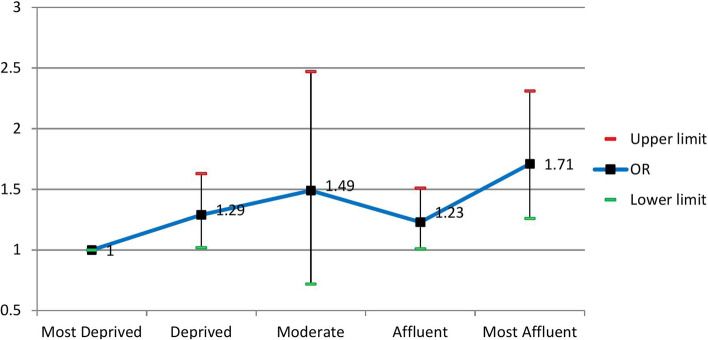
Adjusted ORs (95% CI) of MetS related to the Townsend index

The adjusted odds ratios for SES indicators are shown in Figs. 1, 2, 3 and 4. People with skill level 3 had significantly higher odds than participants with skill level 1 [OR = 1.89 (95% CI; 1.07 to 3.35)]. Participants in the most affluent areas had 71% more odds of MetS than those in the most deprived areas [OR = 1.71 (95% CI; 1.26 to 2.31)]. Besides, age, sex, and physical activity were independently associated with the condition, while no association was seen between cigarette and alcohol use with the disease. The odds ratio of MetS for the age group of ≥ 65 was two times higher than the age group of 35–39 as the reference group [OR = 2.02 (95% CI; 1.24 to 3.27)]. The odds of having MetS in females were 87% higher than in males [OR = 1.87 (95% CI; 1.43 to 2.44)]. Also, the odds ratio of MetS was 57% higher among participants with low physical activity than participants with high physical activity [OR = 1.57 (95% CI; 1.26 to 1.96)].

## Discussion

The results showed significant associations between skill level and Townsend index with MetS, so the people with skill level 3 had significantly higher odds of MetS than those with skill level 1. Also, the participants in the most affluent areas had more odds of MetS than those in the most deprived areas. Besides, the results demonstrated no significant associations between educational level and wealth status with the condition after adjustment for potential confounders.

Our findings showed a considerably high prevalence of MetS in the studied population (39.1%). It was higher than the average rate for Middle Eastern countries (25%) [39]. The prevalence of MetS was higher in women than men. The odds of having MetS was 87% higher in women, concordant with most studies' results [14, 40]. The observed difference can mainly be due to the higher prevalence of important disorders like obesity and diabetes in women compared to men. According to the results, the prevalence of MetS increased with the participants` age. This finding was consistent with most studies conducted in Iran and other countries [14, 18, 41–46].

The prevalence of MetS was significantly higher in smokers compared to non-smokers. In some previous studies, this association was observed [47], while in others, it was not [40, 48]. Also, the prevalence of MetS in alcohol consumers was lower than in non-alcohol consumers (31% vs. 39.3%), but these associations were not significant after controlling for the confounder factors. The effect of alcohol consumption on MetS is inconsistent in the results of various studies. Some studies similar to our results found no relationship between alcohol use and MetS [46, 49]. On the other hand, some studies have shown a positive association between MetS and alcohol consumption [50], while others demonstrated a negative association [51].

According to our results, a significant and inverse association was seen between physical activity and MetS. This finding follows other studies [7, 52, 53]. Several explanations are regarding the beneficial effects of physical activity and Mets, including increased insulin sensitivity [54], better glycemic control via pancreatic β-cell insulin secretory compensation [55], and improved lipoprotein lipids, such as decreased TG and increased high-density lipoprotein cholesterol (HDLC) [56].

Studies on the association between socioeconomic status and diseases are prevalent. In this study, we examined four important indicators that determine economic status. Education is a good indicator of social position in epidemiological studies. It is often seen as the easier way of measuring present socioeconomic status because it precedes other indicators, such as income or occupational-based social position. The univariate results showed a significant inverse relationship between education level and MetS. The illiterate group had the highest prevalence, but this relationship was not significant after controlling the confounders in multivariable regression analysis. This finding is consistent with some results from other studies [47, 57].

Household wealth includes income, savings, and all marketable assets because it captures all household members' personal and intergenerational capital and assets over time. Continuing material security associated with wealth can benefit health through a sense of protection, autonomy, and prestige [58]. In our study, participants in higher wealth quintiles had more MetS than the poorest groups. However, the relationship between the wealth index and MetS was not statistically significant. Conversely, in a similar study, the relationship between the wealth index and MetS was statistically significant. They reported that the prevalence of MetS in the poorest group was higher than in the other groups [59].

After adjusting for confounder factors, our study results showed a statistically significant relationship between skill level and MetS. The results indicated that the odds of having MetS were lower in skill levels one and four. These can be due to higher physical activity and less obesity in participants with skill level one, namely manual workers, and greater awareness about risk factors and a more proper lifestyle in managers belonging to skill level four. In a similar study, the occupational subgroups, equipment, machine operation, and assembling workers group showed the highest prevalence of the MetS, the same as our finding. Still, unlike our results, "managers" had a high rate of MetS. Also, similar to our study, they demonstrated that manual workers were significantly less likely to have the MetS than non-manual workers [60].

In this study, the inverse relationship between the Townsend deprivation index and MetS was statistically significant in multiple logistic regression analyses. In contrast to our findings, Yanan Qiao [61] showed that the Townsend deprivation index was strongly associated with a MetS so that in the residences of the most deprived area, the prevalence of MetS was higher in the most affluent area.

Strengths of our study included; first, the large sample size in the context of a population-based cohort study was one of the strengths of our study that led to more precise estimates by reducing the probability of random error. Second, the use of well-trained questioners and the presence of several levels of supervisors. Third, in this analysis, we applied several indicators of SES at the individual, household, and area levels that can assess different aspects of this complex issue. Fourth, we used valid measures for the diagnosis of MetS that can reduce the probability of the outcome misclassification. Fifth, our study was conducted on the Arab population, ethnicity, and lifestyle of Iranian Arabs, almost the same as those of neighboring countries, especially south of Iraq and Kuwait. So, the findings from the HCS can be generalized to a wide geographical area covering millions of people. On the other hand, there were some limitations in our study. First, the observed associations are not proving causality because the study design was a cross-sectional survey, and reverse causality bias could occur. Another limitation was recruiting prevalent cases instead of incident cases in our study. It can induce selection bias because the assessed patients were only the cases that survived and did not include all the patients. Therefore, the findings of this study may be only generalized to this group of patients and not to fatal cases of the disease.

## Conclusion

In summary, this study showed that SES is related to MetS. Among the four assessed SES indicators, skilled levels and Townsend score are strongly associated with MetS. Also, the older age group, female gender, and low physical activity were other risk factors for MetS. We recommend considering people’s SES when interventional programs are planned and conducted on MetS in similar communities.

## Supplementary Information

**Additional file 1: Table S1.** Prevalence rates of MetS (CI 95%) by sex and age groups. Description of data: We have described the prevalence and its confidence interval in different age groups by gender in the table of Supplementary 1.

## Data Availability

The datasets provided during and/or analyzed during this study are available from the corresponding author upon reasonable request.

## Abbreviations

MetS: Metabolic syndrome
SES: Socioeconomic status
HICs: High income countries
LMICs: Low-and middle-income countries
ORs: Odds ratios
95%CI: 95% Confidence interval
