# Supplementary material for: Socioeconomic status and metabolic syndrome in Southwest Iran: results from Hoveyzeh Cohort Study (HCS)
Source: BMC Endocr Disord. 2022 Dec 28;22:332. doi: 10.1186/s12902-022-01255-5 (PMC9795639; doi:10.1186/s12902-022-01255-5)
Supplement: Supplementary file 1 — Additional file 1: Table S1. Prevalence rates of MetS (CI 95%) by sex and age groups. Description of data: We have described the prevalence and its confidence interval in different age groups by gender in the table of Supplementary 1. [file 12902_2022_1255_MOESM1_ESM.docx]

Supplementary Material

Supplementary Table

**Table S1.** Prevalence rates of MetS (CI 95%) by sex and age groups

| **Age group** | **Male** | | | **Female** | | | **P-value** |
| --- | --- | --- | --- | --- | --- | --- | --- |
|  | n | case | Prevalence (%)  (95% CI) | n | case | Prevalence (%)  (95% CI) |  |
| 35-39 | 732 | 160 | 21.9 (18.9-25.0) | 1180 | 315 | 26.7 (24.2 – 29.3) | 0.017 |
| 40-44 | 794 | 202 | 25.4 (22.4-28.6) | 1231 | 444 | 36.1 (33.4 – 38.8) | <0.001 |
| 45-49 | 704 | 216 | 30.7 (27.3-34.2) | 1093 | 500 | 45.7 (42.8 – 48.8) | <0.001 |
| 50-54 | 608 | 205 | 33.7 (30.0-37.6) | 874 | 467 | 53.4 (50.1 – 56.8) | <0.001 |
| 55-59 | 541 | 186 | 34.4 (30.4-38.6) | 740 | 455 | 61.5 (57.9 – 65.0) | <0.001 |
| 60-64 | 361 | 121 | 33.5 (28.7-38.6) | 437 | 270 | 61.8 (57.0 – 66.4) | <0.001 |
| ≥65 | 286 | 90 | 31.5 (26.1-37.2) | 428 | 282 | 65.9 (61.2 – 70.4) | <0.001 |
| Total | 4026 | 1180 | 29.3 (27.9 – 30.7) | 5983 | 2733 | 45.7 (44.4 – 47.0) | <0.001 |
